# Nutritional Supplement Use in a UK High-Performance Swimming Club

**DOI:** 10.3390/nu15153306

**Published:** 2023-07-26

**Authors:** Josh W. Newbury, S. Andy Sparks, Matthew Cole, Adam L. Kelly, Lewis A. Gough

**Affiliations:** 1 Research Centre for Life and Sport Science (CLaSS), School of Health Sciences, Birmingham City University, Birmingham B9 3TN, UK; josh.newbury@mail.bcu.ac.uk (J.W.N.); matthew.cole@bcu.ac.uk (M.C.); adam.kelly@bcu.ac.uk (A.L.K.); 2 Sport Nutrition and Performance Research Group, Department of Sport and Physical Activity, Edge Hill University, Ormskirk L39 4QP, UK; sparksa@edgehill.ac.uk

**Keywords:** supplements, sport nutrition, ergogenic aids, swimming, adolescent athletes

## Abstract

The aim of this study was to observe the nutritional supplement practices of highly trained swimmers on a national talent pathway, since it is often reported that swimmers engage in widespread supplement use at the elite level. Thus, this study employed a validated supplement intake questionnaire with forty-four swimmers from a high-performance swimming club, which had three distinct talent stages: development (aged 11–14 years, *n* = 20), age-group (aged 13–17 years, *n* = 13), and national level (aged ≥ 16 years, *n* = 11). Ninety-eight percent of the interviewed swimmers reported using at least one supplement, with performance (34%) and recovery (19%) cited as the primary reasons. National swimmers used more total supplements (8.1 ± 3.4 supplements) compared to age-group (4.8 ± 2.0 supplements, *p* = 0.003, *g* = 1.17) and development (3.9 ± 1.7 supplements, *p* < 0.001, *g* = 1.69) swimmers, mostly because of a greater intake of ergogenic aids (2.4 ± 1.4 supplements vs. age-group: 0.5 ± 0.5 supplements, *p* < 0.001, *g* = 1.12; vs. development: 0.1 ± 0.2 supplements, *p* < 0.001, *g* = 1.81). Parents/guardians were the primary supplement informants of development swimmers (74%, *p* < 0.001, *V* = 0.50), whereas performance nutritionists informed ~50% of supplements used by age-group and national swimmers (*p* < 0.001, *V* = 0.51). Based on these results, supplement education and greater focus on basic sport nutrition practices may be required for parents/guardians at the development level. Moreover, further research is needed to support the high number of ergogenic aids used by national swimmers, with the efficacy of these supplements currently equivocal in the applied setting.

## 1. Introduction

The International Olympic Committee (IOC) define nutritional supplements as foods, food components, nutrients, or non-food compounds that are purposely ingested in addition to the habitual diet to achieve a specific health and/or performance outcome [1]. This broad definition describes a wide variety of commercially available supplements. Therefore, Garthe and Maughan [2] recommend the following subcategories: (a) ‘sports supplements’ that provide convenient sources of energy and macronutrients for when whole food sources are impractical (e.g., sports drinks, whey protein); (b) ‘medical supplements’ that can be used to treat and/or avoid clinical issues (e.g., iron, vitamin D_3_); and (c) ‘ergogenic aids’ that have potential to improve exercise performance (e.g., caffeine, creatine monohydrate). There are also other supplements described as ‘herbal’, ‘botanical’, or ‘functional’ that are claimed to optimise health; however, the safety and efficacy of such supplements is questionable for athletes given that their production methods and active ingredients are often unclear [3]. Nonetheless, supplements from each of these categories offer practical benefits that are attractive to athletes, resulting in widespread use across sports at the elite [4], junior [5], and recreational levels [6].

Supplement use generally increases with age and training status, but the total number and type of supplements that are consumed may also be influenced by a sport’s cultural norms [1]. In Olympic athletes, for example, swimmers are often placed among the highest supplement users, with 56% of swimmers reporting using supplements at the Atlanta 1996 Games, which increased to 69% of swimmers at Sydney 2000 [7,8], although, this only consisted of vitamins, minerals, and amino acids; therefore, limited information on sports supplements was provided [1]. Indeed, later reports showed that 97–99% of Australian swimmers used nutritional supplements prior to the 1998 and 2009 World Aquatic Championships [4,9], albeit with an increase in the total number (mean increase: +3.3 supplements) and types of supplements (sports: +1.8 supplements, ergogenic: +1.2 supplements) being consumed across the 11-year window [10]. Along with more supplements now being available to swimmers, there is also an increased dissemination of supplement information (or misinformation) occurring via the internet, which appears to inform most supplement practices made by swimmers and their coaches, rather than seeking advice from qualified nutritionists [11,12]. Consequently, large supplement intakes may not be restricted to elite competitors, with national and international swimmers in Spain both reporting an equally prevalent consumption rate (~87%); including little differences in the total number and types of supplements being reported [12]. Given that adolescents make up a large proportion of competitors at the national level in swimming, this suggests that supplement use is as similarly widespread in adolescents as it is with adults, though with younger cohorts receiving less guidance on safe practices [13]. However, little information is available with this cohort, especially within a high-performance UK landscape. As a result, identifying the current practices of adolescent swimmers may assist with swimmers becoming safe users of supplements as adults and improve their performance as at this stage.

The supplement use of adolescent swimmers is currently difficult to determine as this population’s intakes have either been investigated alongside other sports [11,13] or have been overlooked in dietary investigations [14,15,16]. However, studies that have attempted to document the supplement beliefs and behaviours of highly trained young athletes reported that 35–100% regularly consume nutritional supplements, with the most common reasons being to improve performance (20–65%), enhance recovery (33–40%), and support health (23–56%) [5,11,13,17,18]. Such widespread supplement use can be justified at this age since a ‘food first, but not always food only’ approach to nutrition is optimal for the health and performance of highly trained athletes, even in adolescents where the intake of whole foods around training and competitions can be impractical [19]. Though, what is more dubious is the intake of ergogenic aids as the possibility of marginal performance benefits in this cohort is likely outweighed by the chance of adverse side-effects and/or inadvertent doping [20,21]. Nonetheless, 57–72% of adolescents believe performance-enhancing supplements to be important for sporting success, continuing to use them despite knowing the inherent risks and being unclear on correct dosing protocols [5,11,13], which might be because supplement knowledge is often obtained from coaches and family members, as opposed to qualified nutrition practitioners [5,11,12,13,18,19]. These potential outcomes can provide evidence for monitoring and intervention strategies for supplement education that sport scientists/nutritionists can employ when working with swimmers. As a result, this might improve the athletes’ relationship with supplement usage and, therefore, increase safe practices and remove some potential negative consequences of supplement usage (e.g., inadvertent doping).

The aim of this study was therefore to observe the current supplement practices at three distinct talent stages within a UK-based high-performance swimming club: development phase (aged 11–14 years); national age-group level (aged 13–17 years); and experienced national competitors (aged ≥ 16 years).

## 2. Materials and Methods

### 2.1. Participants

A total of 44 swimmers across three talent stages voluntarily participated in this study (Table 1). The first talent stage (development) consisted of 20 swimmers who were all on a development pathway with the investigated swimming club. All swimmers in this group were aged 11–14 years and all competed at the regional level, with ten swimmers also being nationally ranked in the top 10 swimmers in their respective age groups for at least one event. The second talent stage (age-group) consisted of 13 swimmers who were all nationally competitive in age-group swimming categories (aged 13–17 years) but were not yet at the performance level to consistently qualify for national competitions. Seven of this group were nationally ranked in the top 10 swimmers for their specialist event, whereas one had recently been selected to represent their nation at the junior level. The final talent stage (national) consisted of 11 swimmers who all consistently qualified for national swimming competitions. All members of this group were aged ≥ 16 years, including three national medallists and five swimmers who had represented their respected nations at junior international competitions. An a priori power calculation with input parameters of α = 0.05 and ß = 0.80 determined this sample size appropriate for detecting medium effect sizes (0.50) in one-way analysis of variance tests (three groups, one measure) with a power of 80% (G*Power, v.3.1.9.4, Universität Düsseldorf, Germany) [22].

### 2.2. Experimental Procedures

A descriptive and cross-sectional design was used to observe supplement practices. Each swimmer underwent a short interview (10–15 min) with the lead researcher based on the questions adapted from a validated supplement intake questionnaire [20], which had recently been used to observe supplement use in both competitive pool [12] and open water disciplines [23]. The interview method was used as opposed to an electronic questionnaire due to its logistical ease in adolescent swimmers, as it enabled the researcher to instantly clarify questions and/or ask for further information if answers were unclear. In addition, the researcher also provided a comprehensive list of supplements and was able to explain each one in further detail to facilitate supplement recall. Some adolescents also did not have access to their own mobile devices. Therefore, this method enabled swimmers to answer questions honestly without being influenced by parents/guardians or coaches. The questions that were asked were as follows: (a) what supplements are consumed; (b) why is that supplement consumed; (c) where was the information for that supplement sourced; (d) how frequently is that supplement consumed; and (e) where was the supplement purchased? Swimmers were asked to detail the supplements they consumed in the last 12 months as per previous research [4,12].

Most swimmers and their parents/guardians received sport nutrition support prior to this study, which was embedded into the training schedule by the high-performance swimming club. National swimmers received individual support via nutrition consultations, body composition analysis, competition planning, and advice regarding ergogenic supplementation. Both national and age-group swimmers received classroom-based group education workshops, i.e., [24], were given regular nutritional prompts via mobile group communication (WhatsApp, Menlo Park, CA, USA), and had access to electronic PDF and presentation resources (Google Drive, Mountain View, CA, USA). The nutritionist did not directly engage with development swimmers, but parents/guardians of all three groups received access to online resources and could communicate with the nutritionist via mobile group communication. The level and frequency of individual support provided to swimmers and parents/guardians were determined by their engagement with the provisions.

### 2.3. Data Groups for Analysis

Supplement intakes were compared by talent stage (national vs. age-group vs. development) rather than participant age due to the differing levels of nutrition support they received. For each individual question, the following categories were applied for data analysis based on the swimmers’ responses. *Supplement type*: sports supplements; ergogenic aids; and health supplements, whereby ‘health’ supplements included vitamins, minerals, medical, and herbal supplements. *Reasons for use*: increase performance (including ‘increasing energy levels for racing’); enhance recovery; general health; convenient source of nutrients; muscle growth; immune support (avoiding or reducing length of illnesses); hydration; sleep support; and unsure. *Information source*: performance nutritionist; swim coach; other coach (i.e., physiotherapist, personal trainer); teammate; medical doctor; parent/guardian; friends and siblings; media (i.e., internet, social media role models); and national governing bodies (i.e., recommended at a development camp, provided by supplement partners). *Supplement frequency*: daily; regularly (1–4 days·week^−1^); and occasionally (i.e., at competitions only). *Supplement source*: grocery stores; general stores online (e.g., Amazon); online sport nutrition outlets; health and wellness stores; pharmacies; and supplied directly from a performance nutritionist or a parent/guardian.

### 2.4. Statistical Analysis

All quantitative data (i.e., total supplements, participant characteristics) are presented as mean ± standard deviation, whereas the frequency of the qualitative responses (i.e., individual supplements, information sources, reasons for use) are reported as percentages. Prior to statistical analyses, all data were checked for normality and homogeneity of variance using the Shapiro–Wilk and Levene tests, respectively. Based on the data in this study violating normality and sphericity, non-parametric tests were utilised. Kruskal–Wallis tests were used to analyse group level differences between talent stages (national, age-group, development), whereas Mann–Whitney *U* tests were used to analyse sex-based differences. Hedge’s *g* bias-corrected effect sizes were calculated and reported for pairwise comparisons, based on there being ≤20 participants in each talent group [25]. These effect sizes were interpreted as ‘small’ (0.20–0.49), ‘moderate’ (0.50–0.79), and ‘large’ (≥0.80) [26]. Pearson’s Chi-Square (χ^2^) tests were used to determine differences in frequency distributions between groups. Cramer’s V effect sizes were calculated for comparisons between frequency distributions, which were interpreted as ‘weak’ (0.05–0.09), ‘moderate’ (0.10–0.14), ‘strong’ (0.15–0.24), and ‘very strong’ (≥0.25) [27]. Statistical significance was set at *p* < 0.05, and all statistical analyses were performed using the Statistical Package for Social Sciences (v.28, IBM Statistics, Chicago, IL, USA).

## 3. Results

### 3.1. Participants

There were incremental increases in age (*p* < 0.001) and years competitive (*p* < 0.001) between the training phases (Table 1), such that national swimmers were older and more experienced than age-group swimmers (*p* < 0.001, *g* = 3.14; and *p* < 0.001, *g* = 1.73, respectively), and age-group swimmers were older and more experienced than development swimmers (*p* < 0.001, *g* = 1.95; and *p* < 0.001 *g* =1.73, respectively). Moreover, the swimmers’ mean WA points were also increased at each talent stage (*p* < 0.001), with national swimmers being higher performers compared to age-group swimmers (*p* = 0.010, *g* = 1.14), and age-group swimmers being higher performers than development swimmers (*p* < 0.001, *g* = 1.99). An increased number of weekly training sessions (*p* = 0.008) and training hours (*p* < 0.001) was also observed, but only between swimmers of development and age-group levels (*p* = 0.005, *g* = 1.44; and *p* < 0.001, *g* = 1.94, respectively). There were no sex-based differences in any participant characteristics (all *p* > 0.05).

### 3.2. Supplement Type and Prevalence

Ninety-eight percent (43 of 44) of swimmers reported using at least one nutritional supplement. These supplement intakes differed between talent stages (*p* < 0.001, Table 2), such that national swimmers reported using a greater number of individual supplements compared to both age-group (*p* = 0.003, *g* = 1.17) and development swimmers (*p* < 0.001, *g* = 1.69). No difference in total supplement intake was observed between development and age-group swimmers (*p* = 0.169, *g* = 0.35).

Group differences in the consumption of ergogenic aids (*p* < 0.001) and health supplements (*p* = 0.011) were also identified; however, all three talent stages reported using a similar number of sports supplements (*p* = 0.982; Table 2). With regards to ergogenic aids, a higher proportion of national swimmers declared the consumption of beta-alanine (*p* = 0.002, *V* = 0.54); caffeine anhydrous (*p* < 0.001, *V* = 0.68); caffeine drinks and gels (*p* = 0.008, *V* = 0.47); creatine monohydrate (*p* < 0.001, *V* = 0.62); and sodium bicarbonate (*p* = 0.001, *V* = 0.55) compared to development swimmers. This resulted in national swimmers reporting more ergogenic aids compared to age-group swimmers (*p* < 0.001, *g* = 1.81), whereas age-group swimmers also reported consuming more ergogenic aids compared to development swimmers (*p* = 0.005, *g* = 1.12). An increased intake of health supplements also occurred in national swimmers compared to both age-group (*p* = 0.032, *g* = 0.79) and development levels (*p* = 0.003, *g* = 1.27). This occurred due to an increased proportion of national swimmers declaring the use of magnesium (*p* = 0.043, *V* = 0.38), omega-3 fatty acids (*p* = 0.003, *V* = 0.51), vitamin D_3_ (*p* = 0.016, *V* = 0.44), and zinc supplements (*p* < 0.001, *V* = 0.62). No difference in health supplement use was found between age-group and development swimmers (*p* = 0.621, *g* = 0.26). Despite a similar number of sports supplements being reported between groups, a larger distribution of sports drinks was identified in development swimmers (*p* < 0.001, *V* = 0.59), whereas protein powders were more frequently reported by national swimmers (*p* = 0.003, *V* = 0.51).

Male and female swimmers both reported using a similar total number of supplements (*p* = 0.085, *g* = 0.45), including a similar number of ergogenic (*p* = 0.484, *g* = 0.07) and health supplements (*p* = 0.103, *g* = 0.44; Table 2). A greater proportion of males did, however, report using multivitamin supplements (*p* = 0.007, *V* = 0.41). Male swimmers also reported using more sports supplements compared to females (*p* = 0.021, *g* = 0.76), with a greater proportion of males consuming protein-enhanced foods (*p* = 0.036, *V* = 0.32). In contrast, a greater proportion of females reported ingesting caffeine anhydrous (*p* = 0.034, *V* = 0.32), whereas males used a wider variety of ergogenic supplements.

### 3.3. Reasons for Supplement Use

**Figure 1:**
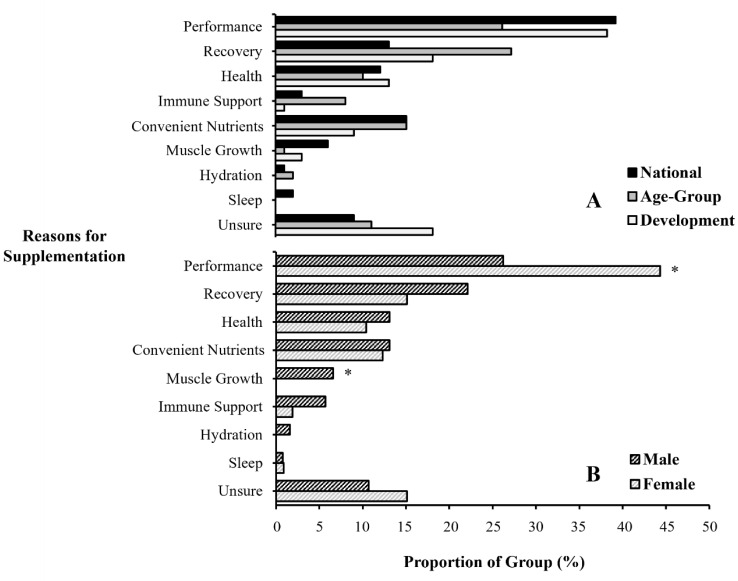
Reasons for nutritional supplement use reported by swimmers in accordance with (**A**) their stage on the national talent pathway; and (**B**) their sex. * Proportional difference between groups (*p* < 0.05).

All three talent stages cited similar reasons for their supplement use (all *p* > 0.05, *V* < 0.15), with ‘performance’ (34 ± 7%) and ‘recovery’ (19 ± 7%) being the largest motivators (Figure 1A). Other popular reasons for supplement consumption were for ‘convenient nutrient sources’ (13 ± 3%) and for ‘general health’ (12 ± 2%). Out of all supplements used, swimmers stated that they were ‘unsure’ why they consumed 18 ± 5% of their total supplements. The reasons for supplement use differed between sexes, such that female swimmers consumed more supplements for ‘performance’ (*p* = 0.003, *V* = 0.20), whereas male swimmers consumed more supplements for ‘muscle growth’ (*p* = 0.007, *V* = 0.18) (Figure 1B). No other sex-based differences occurred (all *p* > 0.05, *V* < 0.10).

### 3.4. Information Sources

Seventy-five percent of all reported supplements were informed by a parent/guardian or a performance nutritionist, though this distribution was not equal across groups (Table 3). Development swimmers were most reliant on parent/guardian information, whose influence was less present in national swimmers (*p* < 0.001, *V* = 0.50). In contrast, national and age-group swimmers both reported a performance nutritionist as the most influential supplement advisor, compared to no swimmers at the development level (*p* < 0.001, *V* = 0.51). Additionally, a greater proportion of national swimmers reported gaining supplement information from their swimming coach compared to the other training stages (*p* = 0.007, *V* = 0.21), whereas the development group sourced more information from the media (*p* = 0.012, *V* = 0.20). A sex-based difference was also found, whereby a greater proportion of female swimmers sourced their supplement information from a performance nutritionist (*p* < 0.001, *V* = 0.29), compared to males who sourced more information from a parent/guardian (*p* = 0.010, *V* = 0.17) and other coaches (*p* = 0.050, *V* = 0.13).

### 3.5. Supplement Frequency

The frequency that swimmers consumed nutritional supplements differed by talent stage (*p* < 0.001). This change occurred as national swimmers reported consuming 3.8 ± 1.8 supplements on a daily basis, which was more than the swimmers at the age-group (1.8 ± 1.6 supplements, *p* = 0.010, *g* = 1.17) and development levels (0.9 ± 0.9 supplements, *p* < 0.001, *g* = 2.20). No further differences were identified between groups for the number of supplements consumed regularly (national: 2.4 ± 1.7; age-group: 1.7 ± 1.2; development: 1.5 ± 0.9 supplements; *p* = 0.255) or occasionally (national: 1.9 ± 1.3; age-group: 1.3 ± 0.6; development: 1.6 ± 0.9 supplements; *p* = 0.526). Between sexes, males reported using more supplements than females on a daily (2.3 ± 1.6 vs. 1.4 ± 1.9 supplements; *p* = 0.033, *g* = 0.49) and regular basis (2.1 ± 1.1 vs. 1.4 ± 1.3 supplements; *p* = 0.045, *g* = 0.52), but no differences occurred in occasional supplements use (1.4 ± 0.9 vs. 1.7 ± 1.0 supplements; *p* = 0.226, *g* = 0.37).

### 3.6. Supplement Sources

**Figure 2:**
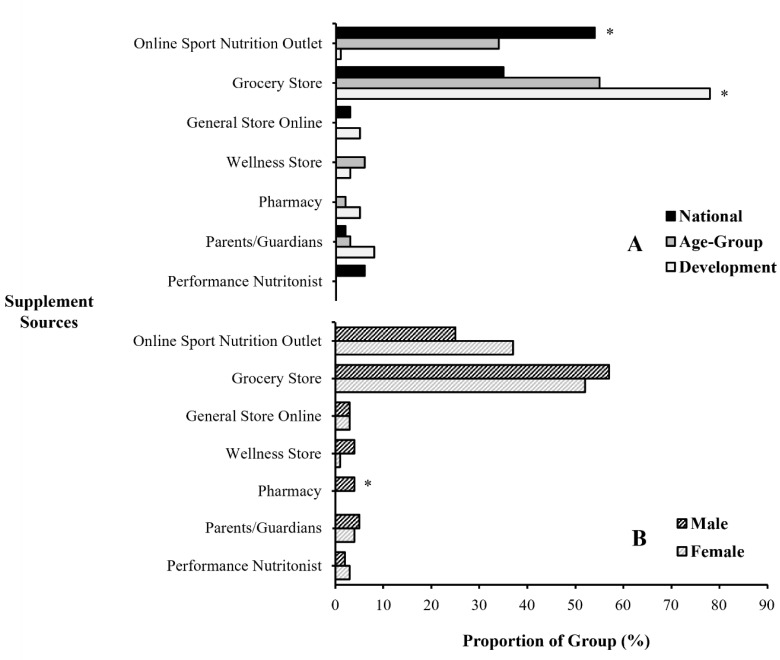
The supplement information sources reported by swimmers within a high-performance swimming club based on (**A**) their stage on the national talent pathway; and (**B**) their sex. * Proportional difference between groups (*p* < 0.05).

Within national swimmers, an increased proportion of supplements were purchased from online sport nutrition outlets (*p* < 0.001, *V* = 0.76), whereas development swimmers purchased most supplements from grocery stores (*p* < 0.001, *V* = 0.35) (Figure 2B). National swimmers were also the only group to source supplements directly from a performance nutritionist (*p* = 0.002, *V* = 0.24), which were all ergogenic aids. No sex differences were observed for most supplement sources (all *p* > 0.05, *V* < 0.10), though three male swimmers were clinically prescribed iron from a pharmacy, leading to a proportional difference compared to female swimmers (*p* = 0.035, *V* = 0.14).

## 4. Discussion

The key finding from this study was that swimmers of all talent stages engaged in widespread supplement use, with swimmers at the development phase (aged 11–14 years) utilising sports supplements at competitions and national swimmers (aged ≥ 16 years) using an array of health and ergogenic supplements on a more regular basis. Indeed, national swimmers reported consuming a similar number of ergogenic and health supplements as international-level adult swimmers in previous research [4,12]. Moreover, swimmers from all three training stages reported ‘performance’ as a key motivator for supplement use, which was in accordance with other swimming cohorts [12,23]. The prevalent use of ergogenic aids in this study was likely due to swimmers having increased access to sport nutrition support as they progressed in training status, as evidenced since parents/guardians were displaced by performance nutritionists as supplement informers in swimmers of age-group and national levels. It is therefore prudent to suggest that supplement education could be best implemented to parents/guardians at the development stage to facilitate safe and effective supplement use later in the swimming career.

Development swimmers all reported using nutritional supplements, with approximately four different supplements being used across a swimming season (~1 daily, ~1–2 regularly, ~1–2 occasionally). Sports drinks were used most frequently (95%), followed by multivitamins (50%) and protein supplements (40–45%), which was comparable to the supplement use in adult and adolescent swimmers of international competitive status (sports drinks: 92–100%, multivitamins: 32–46%, protein powder: 46–58%) [4,28]. This early use of supplements was mostly informed by parents/guardians (74%), who were responsible for purchasing supplements and often supplied them to swimmers without rationale (swimmers were unsure why they consumed 18% of all supplements). The primary motivation for supplement use in this cohort was performance (38%), though it was unclear whether this rationale was led by parents/guardians or influenced by swimmers while shopping with parents/guardians at grocery stores. However, the ‘performance supplements’ reported by this cohort were mostly from the sports supplement category (96%) as opposed to ergogenic aids (4%), which was appropriate at this training age given that sports supplements carry lower risks of side-effects and/or inadvertent doping [2]. This outcome may therefore be viewed positively as swimmers and their parents/guardians identified nutrition as an important factor for swimming performance. Though, this should be interpreted cautiously as a performance nutritionist provided resources to parents/guardians at the development level in this study, whereas other swimming parents/guardians might rely on supplement information from coaches and the internet [11,12,18]. As such, it is difficult to generalise the outcomes of this study to the wider swimming community at present, requiring further investigation across multiple swimming clubs with varying levels of nutrition support.

Age-group swimmers consumed a similar number of supplements as development swimmers (~5 per swimmer), albeit with a change in their supplement choices, reasoning, and information sources. Indeed, the percentage of swimmers using pill and powder sports supplements was increased compared to the development group (electrolytes: +33%, protein powder: +42%), whereas more swimmers also consumed ergogenic aids (46 vs. 5%). The use of caffeine (23%), creatine monohydrate (8%), beta-alanine (8%), and beetroot juice (8%) was reported, with each of them having strong evidence of a performance-enhancing effect [1], and they were consumed in equal proportion to highly trained adolescents from mixed sporting backgrounds (i.e., caffeine: 19%, creatine monohydrate: 25%, beta-alanine: 5%, nitrates: 3%) [11]. However, despite declaring more ergogenic aids, less age-group swimmers used supplements for ‘performance’ compared to development swimmers (−12%), instead citing ‘recovery’, ‘immunity’, and ‘convenience’ as motivating factors. This change was partly due to the introduction of formal sports nutrition education, with a performance nutritionist appearing to replace parents/guardians as the primary source of supplement information (performance nutritionist: +50%, parent/guardian: −34% vs. development swimmers). In turn, this may have enabled age-group swimmers to provide more appropriate reasons for their supplement use. Based on these findings, a transitional stage in supplement use was identified, whereby age-group swimmers become more exposed to sport nutrition and begin trialling ergogenic aids. It is therefore imperative that a ‘performance-enhancing’ diet is not undermined at this age, which may be supported by practical workshops that develop food literacy and cooking skills [29]. Furthermore, strong anti-doping messages would also be of benefit, informing swimmers and guardians of the risks of inadvertently ingesting banned substances when using pill and powder nutritional supplements [2].

National swimmers used an average of eight different nutritional supplements (~3 health, ~3 ergogenic, ~2 sports), which was in line with previous observations in international- and national-level swimmers (6–10 individual supplements) [4,12]. This was, however, higher than the total number of supplements used by age-group swimmers, most notably since more swimmers reported using ergogenic aids (creatine monohydrate: +47%, beta-alanine: +37%) and health supplements (omega-3 fatty acids: +55%, vitamin D_3_: +35%) on a daily basis. Moreover, national swimmers used more ergogenic aids than age-group swimmers at competitions (caffeine: +59, sodium bicarbonate: +36%), some of which were directly sourced from a performance nutritionist. These supplement behaviours follow the step-like process outlined by Garthe and Maughan [2], whereby an increased training status is accompanied by increased access to professional competence, the use supplements to enhance training adaptations, and more tailored use of ergogenic aids for competition. However, performance nutritionists in this study were of greater influence compared to previous observations in national and international swimmers (51% vs. 20–36%) [4,12]. Indeed, previous studies in swimmers and other highly trained young athletes typically cite their coach as the main supplementation informant (38–40%) [11,12], which was in contrast to the present study (12%). This could indicate that sport nutrition support was more prioritised at the investigated swimming club than in others, making it unclear if these supplement practices can be generalised to the wider UK swimming population. In addition, the national swimmers in this study utilised a large number and variety of ergogenic supplements despite the fact that they were yet to have strong evidence specifically in applied swimming settings, thus supporting the need for further supplement research within this population.

Males and females both utilised a similar number of nutritional supplements, although there were sex-based differences in the individual supplements that were used. Female swimmers were more likely to state ‘performance’ as their primary reason for using supplements (+18% vs. males) and engaged in a greater use of caffeine anhydrous (+29% vs. males). However, this was likely due to the national group of swimmers consisting of a larger proportion of females (8 of 11 swimmers), meaning that more females would have been receiving ergogenic supplement support directly from a performance nutritionist. In contrast, male swimmers utilised more protein-based supplements (enhanced foods: +32%, bars: +17%, powder: +13% vs. females) and multivitamin preparations (+40% vs. females), resulting in male swimmers using more supplements on a daily and regular basis. This was in combination with more male swimmers reporting the use of supplements for ‘muscle gain’ (8% vs. no females), which was in accordance with previous investigations in young athletes [11,30,31]. In all, since there were little differences in the supplement behaviours of male and female swimmers, these results suggest that sex-based supplement education is not required.

A limitation of this study was that supplement interviews were based on a previously validated supplement intake questionnaire that was originally produced and validated for mass dissemination within the Spanish sports population [32]. This therefore limited the number of questions that were included, meaning that participants did not elucidate any information regarding dosing strategies or whether supplements were sourced from batch-tested suppliers, both of which are important considerations for determining whether supplements are being consumed in a safe and effective manner [2]. It is also worth noting that the data collected in this study are from one high-performance club in the UK, and therefore, further clubs could have been recruited (i.e., this would also improve the sample size in the study). Equally, we acknowledge that the sample size priori power calculation was not met for the national group in this study (*n* = 11 vs. *n* = 14). It is important, therefore, to interpret these findings with caution. However, it is important to note that sample size determination is not only determined by a prior power calculation, and factors such as resource constraints, accuracy, and heuristics are also important [33]. Moreover, given the difficulty of recruiting high performance athletes, the lack of athletes in this population, and the time-constraints on research, this was not possible for this study. This could have been overcome with the currently used interview method; hence, future investigations should consider utilising this approach with more in-depth questioning surrounding the use of each individual supplement. Nonetheless, the current methods were sufficient to appropriately identify general supplement practices in swimmers of different training status.

In summary, swimmers were identified as prevalent users of nutritional supplements from the development age (aged 11–14 years) through to those performing consistently at the national level. Development swimmers’ supplement practices were largely influenced by parents/guardians, resulting in many sports supplements being consumed for the purpose of ‘performance enhancement’. However, increased access to sport nutrition support was granted at the age-group (aged 13–17 years) and national (aged ≥ 16 years) levels, which subsequently led to the influence of parents/guardians being displaced by performance nutritionists. It was at these talent stages that a greater uptake of ergogenic aids was identified, likely requiring targeted nutrition interventions at the age-group and national levels to ensure safe practices are being followed. Moreover, since many ergogenic aids were used without much supporting evidence, further research in applied swimming settings is required to understand which, if any, of these supplements can benefit the training and/or competitive performances of highly trained adolescent swimmers.

## Figures and Tables

**Table 1:** Participant competitive status and training characteristics.

Measure	National (*n* = 11)	Age-Group (*n* = 13)	Development (*n* = 20)	Males (*n* = 21)	Females (*n* = 23)	Combined (*n* = 44)
Age (years)	20 ± 2	15 ± 1	13 ± 1	16 ± 3	15 ± 3	15 ± 3
Years competitive	9 ± 2 *^#^	5 ± 1 *	3 ± 1	5 ± 3	5 ± 3	5 ± 3
Weekly training sessions	6.9 ± 1.2 *	6.2 ± 0.8 *	5.7 ± 0.5	6.0 ± 0.7	6.3 ± 1.1	6.2 ± 0.9
Weekly training hours	17.6 ± 3.2 *	15.8 ± 2.4 *	12.3 ± 1.2	14.4 ± 2.3	14.9 ± 3.8	14.6 ± 3.1
WA points	698 ± 59 *^#^	622 ± 67 *	483 ± 69	555 ± 123	598 ± 99	578 ± 112

World Aquatic (WA) points awarded for swimmers’ fastest long-course (50 m) swimming performance in 2022. Training hours include both pool and land activities undertaken at the swimming club. * Greater compared to development (*p* < 0.05). ^#^ Greater compared to age-group (*p* < 0.05). Statistical comparisons are described in Section 3.1.

**Table 2:** Total number and prevalence of nutritional supplements reportedly used by three training tiers within a UK-based, high-performance swimming club.

Category/Individual Supplements	Overall (*n* = 44)	National (*n* = 11)	Age-Group (*n* = 13)	Development (*n* = 20)	Males (*n* = 21)	Females (*n* = 23)
Total (supplements)	5.2 ± 2.9	8.1 ± 3.4 ^ab^	4.8 ± 2.0	3.9 ± 1.7	5.9 ± 2.7	4.6 ± 2.9
Sports (supplements)	2.5 ± 1.0 *^#^	2.7 ± 1.7	2.6 ± 0.7	2.6 ± 0.9	2.9 ± 0.8 ^†^	2.2 ± 1.0
Dextrose/maltodextrin (%)	0	0	0	5	0	4
Electrolytes (%)	18	18	38	5	29	9
Liquid meals (%)	9	18	0	10	0	17 ^‡^
Protein bars (%)	43	36	54	40	57	30
Protein-enhanced food (%)	45	45	46	45	62 ^‡^	30
Protein powder (%) ^†^	45	82	54	20	52	39
Sports bars (%)	2	0	0	5	0	4
Sports drinks (%) ^†^	68	27	62	95	67	70
Sports gels (%)	18	9	8	30	19	17
Ergogenic (supplements)	0.8 ± 1.4	2.4 ± 1.4 ^ab^	0.5 ± 0.5 ^b^	0.1 ± 0.2	0.8 ± 1.7	0.9 ± 1.2
Beetroot juice (%)	5	9	8	0	10	0
Beta-alanine (%) ^†^	14	45	8	0	14	13
Caffeine anhydrous (%) ^†^	30	82	23	5	14	43 ^‡^
Caffeine drinks/gels (%) ^†^	9	36	0	0	10	9
Citrulline malate (%)	2	9	0	0	5	0
Creatine monohydrate (%) ^†^	16	55	8	0	19	13
Sodium bicarbonate (%) ^†^	9	36	0	0	10	9
Health (supplements)	1.8 ± 1.6 *	3.0 ± 1.3 ^ab^	1.7 ± 1.8	1.3 ± 1.3	2.2 ± 1.4	1.5 ± 1.7
Ginger (%)	2	0	8	0	5	0
Iron (%)	20	27	15	20	24	17
Magnesium (%) ^†^	5	18	0	0	10	0
Melatonin (%)	2	9	0	0	0	4
Multi-vitamin (%)	41	36	31	50	62 ^‡^	22
Omega-3 fatty acids (%) ^†^	20	55	0	15	19	22
Probiotics (%)	20	9	38	15	24	17
Vitamin C (%)	18	27	31	5	24	13
Vitamin D_3_ (%) ^†^	39	73	38	20	38	39
Zinc (%) ^†^	11	45	0	0	10	13

* Greater intake compared to health supplements (*p* < 0.05); ^#^ greater intake compared to ergogenic aids (*p* < 0.05); ^a^ greater intake compared to age-group swimmers (*p* < 0.05); ^b^ greater intake compared to development swimmers (*p* < 0.05); ^†^ = percentage difference between talent stages (*p* < 0.05); ^‡^ = percentage difference between sexes (*p* < 0.05).

**Table 3:** Distribution (%) of supplement information sources reported by swimmers within a high-performance swimming club.

Information Source	Overall (*n* = 228)	National (*n* = 89)	Age-Group (*n* = 62)	Development (*n* = 77)	Males (*n* = 122)	Females (*n* = 106)
Performance nutritionist (%) *	33	51	50	0	20	48 ^#^
Swim coach (%) *	6	12	3	1	7	5
Parent/guardian (%) *	42	16	40	74	50 ^#^	33
NGB (%)	3	4	2	1	4	1
Medical doctor (%)	3	1	3	4	4	1
Other coach (%)	4	7	0	3	6 ^#^	1
Teammate (%)	3	3	0	4	2	2
Friends and siblings (%)	2	2	0	4	1	0
Media (%) *	3	1	0	8	3	5
Self-Research (%)	2	2	2	1	1	1

* Proportional difference between talent stages (*p* < 0.05); ^#^ proportional difference between sexes (*p* < 0.05); *n* = total number of supplements reported by each group.

## Data Availability

Data sets can be obtained through contacting the corresponding author.
